# Supplementary material for: The Systems Biology Research Tool: evolvable open-source software
Source: BMC Syst Biol. 2008 Jun 29;2:55. doi: 10.1186/1752-0509-2-55 (PMC2446383; doi:10.1186/1752-0509-2-55)
Supplement: Additional file 1 — SBRT Archive. An archive of the current version of the Systems Biology Research Tool. [file 1752-0509-2-55-S1.zip › sbrt-1.4.0/doc/developers_guide/api/sbrt/ext/R/R.html]

R


|  |  |  |
| --- | --- | --- |
| **Overview** \| **Package** \| **Class** \| **Use** \| **Tree** \| **Deprecated** \| **Index** \| **Help** | |  |
| **PREV CLASS**   NEXT CLASS | **FRAMES**    **NO FRAMES**     **All Classes** |  |
| SUMMARY: NESTED \| FIELD \| CONSTR \| METHOD | DETAIL: FIELD \| CONSTR \| METHOD |  |


---


## sbrt.ext.R Class R

```
java.lang.Object
  sbrt.ext.R.R
```

---

``` public final class R extends java.lang.Object ```

This class is used to access the R engine safely. To use the engine,
it must first be locked. This grants sole access to the one holding the
key. The key must be supplied for each evaluation. When finished using the
engine, the key-holder should unlock it. The R engine cannot be stopped
after it has been started, except by Runtime.exit(int).

**Author:**
:   This class was written and documented by
    Jeremiah Wright while in the Wagner lab.

---

| **Method Summary** | |
| --- | --- |
| `static org.rosuda.JRI.REXP` | `eval(java.lang.String statement, java.lang.Object key)`             Evaluates the provided R command. |
| `static boolean` | `isKey(java.lang.Object potentialKey)`             Indicates if the provided object is the key to unlock the R engine. |
| `static boolean` | `isLocked()`             Indicates if the R engine is currently locked. |
| `static java.lang.Object` | `lock()`             Locks the R engine so that no one can use it without the key. |
| `static void` | `startR()`             Starts the R engine if it is not already running. |
| `static void` | `unlock(java.lang.Object key)`             Unlocks the R engine so that others have access, that is, someone can request a new key. |

**Methods inherited from class java.lang.Object**

`clone, equals, finalize, getClass, hashCode, notify, notifyAll, toString, wait, wait, wait`

**Method Detail**

### isLocked

```
public static boolean isLocked()
```

:   Indicates if the R engine is currently locked.

    :   **Returns:**: `true` if the R engine is locked, `false` othewise.

---


### isKey

```
public static boolean isKey(java.lang.Object potentialKey)
```

:   Indicates if the provided object is the key to unlock the R engine.

    :   **Parameters:**: `potentialKey` - the potential key object. **Returns:**: `true` if the provided object can unlock the R engine, `false` otherwise.

---


### lock

```
public static java.lang.Object lock()
```

:   Locks the R engine so that no one can use it without the key.

    :   **Returns:**: the key that can unlock the R engine.

---


### unlock

```
public static void unlock(java.lang.Object key)
```

:   Unlocks the R engine so that others have access, that is, someone can
    request a new key. If the provided key does not fit the current lock,
    no action is taken.

    :   **Parameters:**: `key` - the key that fits the current lock.

---


### eval

```
public static org.rosuda.JRI.REXP eval(java.lang.String statement,
                                       java.lang.Object key)
```

:   Evaluates the provided R command.

    :   **Parameters:**: `statement` - the R command to be evaluated.: `key` - the key required to unlock the R engine. **Returns:**: the result of executing the provided R command. **Throws:**: `java.lang.IllegalArgumentException` - if the provided key does not fit the lock.: `JriException` - if the R engine throws an exception.

---


### startR

```
public static void startR()
```

:   Starts the R engine if it is not already running. It is not necessary to
    call this method, because it is called automatically when required. If the
    R engine is already running, no action is taken.

    :   **Throws:**: `JriException` - if an exception is thrown while attempting to start the R engine.


---


|  |  |  |
| --- | --- | --- |
| **Overview** \| **Package** \| **Class** \| **Use** \| **Tree** \| **Deprecated** \| **Index** \| **Help** | |  |
| **PREV CLASS**   NEXT CLASS | **FRAMES**    **NO FRAMES**     **All Classes** |  |
| SUMMARY: NESTED \| FIELD \| CONSTR \| METHOD | DETAIL: FIELD \| CONSTR \| METHOD |  |


---
